# A Latent Profile Analysis of Psychosis Symptoms to Examine Distress and Depression as Pathways to Suicide Ideation Among Individuals in an Early Phase of Psychosis Illness

**DOI:** 10.1111/eip.70013

**Published:** 2025-02-18

**Authors:** Lindsay A. Bornheimer, Nicholas M. Brdar, Adrienne Lapidos, Alexandra N. Kelter, Chloe Miner, Andrew Grogan‐Kaylor

**Affiliations:** ^1^ School of Social Work University of Michigan Ann Arbor Michigan USA; ^2^ Department of Psychiatry Michigan Medicine Ann Arbor Michigan USA

**Keywords:** depression, distress, latent profile analysis, psychosis, suicide

## Abstract

**Background:**

Suicide rates are high among individuals in first episode psychosis and there is a critical need to better understand drivers of suicide risk to inform treatment efforts. This study identified profiles of psychosis symptoms and examined a mediation model of depression and distress as mechanisms in the relationships between psychosis symptoms and suicide ideation by latent profiles.

**Methods:**

Data were obtained from the Human Connectome Project for Early Psychosis (*n* = 166) of individuals between 16 and 35 years of age who had onset of affective or non‐affective psychosis within 5 years of consent. Data were analysed using Latent Profile Analysis (LPA) and Structural Equation Modelling in MPlus.

**Results:**

LPA revealed the following groups: (1) relatively lower and more balanced levels of symptoms, (2) highest positive and general symptoms and (3) highest negative symptoms. Findings indicated the relationships in the model differed between by LPA groups. Distress and depression functioned as mediators between psychosis symptoms and suicide ideation for Groups 1 and 2.

**Conclusions:**

A better understanding of the roles that distress and depression play in the relationships between psychosis symptoms and suicide ideation can help inform modifiable targets of early intervention and subsequently decrease risk for suicide.

## 1 Introduction

Schizophrenia spectrum disorders (SSDs) affect over 21 million people globally (Orrico‐Sánchez et al. 2020) and are well‐known to increase the risk of premature death (Harris et al. 2021; Institute of Health Metrics and Evaluation, 2022; Laursen et al. 2014; Walker et al. 2015). Data show that 5%–10% of individuals with SSDs die by suicide in comparison to 0.3% of individuals who do not have a serious mental illness (Holmstrand et al. 2015; Hor and Taylor, 2010; Palmer et al. 2005). Data furthermore show the risk for suicide death among individuals in a First Episode of Psychosis (FEP) is elevated by 60% within the first year of treatment compared to individuals who have been in treatment for more than 1 year (Bornheimer 2019; Bornheimer et al. 2022; Fleischhacker et al. 2014; Nordentoft et al. 2004; Nordentoft, Madsen, and Fedyszyn 2015; Pompili et al. 2011; Courtet 2018). Given the elevated rates of suicide risk in FEP, and well‐documented research of FEP being a critical timeframe for psychiatric intervention (Birchwood, Todd, and Jackson 1998; Bornheimer et al. 2022; Reed 2008), a better understanding of the factors contributing to risk for suicide thoughts and behaviour are needed to inform treatment efforts (Bornheimer et al. 2020).

Depression is a well‐documented contributor to suicide risk and outcomes (i.e., thoughts and behaviour) with data showing that 20%–40% of people with SSDs experience symptoms of depression in a lifetime (Birchwood, Iqbal, and Upthegrove 2005; Conley et al. 2007; Pelizza et al. 2022). Furthermore, depression is known to impact functional and clinical outcomes, and is also strongly related to suicide ideation, attempt and death (Upthegrove, Marwaha, and Birchwood 2017). Sönmez et al. (2016) found that persistent depression in the first year of psychosis treatment in a FEP sample was associated with higher levels of suicide behaviour across 10‐year follow up assessments in comparison to people who did not have persistent depression in their first year of treatment.

Distress is known to contribute to depression (Freeman et al. 2015) and has been shown to arise in relation to positive (e.g., hallucinations and delusions) and negative (e.g., affective flattening and alogia) symptoms among people with SSDs (Garcia‐Mieres et al. 2020; Tsang et al. 2021). People with psychosis are particularly vulnerable to the experience of distress (Mawson, Cohen, and Berry 2010), with data showing negative affect, emotion dysregulation and stress sensitivity function as important factors in distress (Grove et al. 2016; Phalen et al. 2024). It is furthermore theorised that people with psychosis may have limited ability to communicate their distress to others given experiences of negative symptoms, such as affective flattening (Myin‐Germeys et al. 2000), and greater challenges in organising and naming emotions in general (O'Driscoll et al. 2014; Kimhy et al. 2012).

Studies have found suicide experiences in populations with SSDs may arise from psychological distress that emerges in relation to the experience of having hallucinations and delusions (Kelleher et al. 2015; Mawson, Cohen, and Berry 2010; Ventriglio et al. 2016). In fact, research indicates that distress is one of the strongest predictors of suicide thoughts and behaviour among people with psychosis, along with depression and history of suicide attempt (Challis et al. 2013; Hawton et al. 2005). Furthermore, a study by Martin and colleagues (2015) found that psychosis alone did not predict future suicide attempt, but psychosis in combination with psychological distress was a strong predictor. It is possible that negative self‐appraisals may contribute to the experience of hopelessness, defeat and entrapment, with subsequent impact on distress and suicide thinking (Johnson et al. 2010a). This prior research aligns with the Cry of Pain (CoP; Williams 1997) model and Schematic Appraisals Model of Suicide (SAMS; Johnson et al. 2010b; Williams et al. 2005). The CoP theory of suicide is grounded within ecological explanations of stress and defeat including the belief that feelings of extreme and uncontrollable stress are important and necessary components contributing to mechanisms of suicide. Constructs within the theory include the presence of stressors, perception of stressors, cognitive biases, hopelessness, feelings of no rescue from others, and means for suicide (Tarrier et al. 2013). The SAMS builds on components of CoP to and is one of few theoretical models of suicide risk that specifically considers symptoms of psychosis. In particular, SAMS suggests positive self‐appraisals provide a key source of resilience by buffering suicidal thoughts and behaviours (Tarrier et al. 2013).

Given the treatment of depression in FEP populations can improve functional outcomes and reduce suicide risk (Upthegrove, Marwaha, and Birchwood 2017) understandings are critically need of mechanisms involved in the relationship between psychosis symptoms and suicide ideation to inform treatment efforts. Less is specifically known about profiles of psychosis symptoms and how various experiences of positive, negative and general symptoms may condition the ways in which mechanisms of depression and distress impact suicide ideation. Understanding how unique combinations of psychosis symptoms (i.e., profiles of psychosis symptoms) may impact depression, distress and suicide ideation can inform individualised tailoring of treatment efforts and transdiagnostic behavioural measures to be used alongside computational methods in individual‐level prediction and clinical decision making, such as in precision medicine (Passos et al. 2022).

This study aimed to (1) identify distinct profiles of individuals in an early phase of psychosis illness based on latent groupings of psychosis symptoms (positive, negative and general symptoms); and (2) conduct a multiple group analysis of a mediation model to examine differences in depression and distress functioning as mechanisms in the relationship between psychosis symptoms and suicide ideation by the psychosis profile groupings. Overall, we hypothesised that (1) greater positive, negative and general symptoms of psychosis would relate to greater distress and depression, (2) heightened distress would relate to higher levels of depression and (3) greater depression would relate to greater suicide ideation. We furthermore hypothesised that the relationships between psychosis symptoms, depression, distress and suicide ideation would vary across groupings of participants who have different profiles of psychosis symptom experiences.

## 2 Methods

Secondary data were obtained from the Human Connectome Project for Early Psychosis (HCP‐EP). The HCP‐EP collected data similar to the original Young Adult HCP, yet with a focus on young adults who are in an early phase of psychosis (within 5 years of onset). Participants were recruited from 4 clinical sites in the United States. Greater detail of the HCP‐EP can be found in Elam et al. (2021). The current secondary data study was approved by the University of Michigan Institutional Review Board and ethical approval was obtained for the original study by the Harvard Medical School Institutional Review Board.

### 2.1 Sample

A total of 166 individuals were included in the HCP‐EP study with affective and non‐affective psychosis. All study participants were between the ages of 16 and 35 at the time of consent, fluent in English and meeting criteria for affective or non‐affective psychosis with onset being within 5 years. Affective psychosis included major depression with psychosis or bipolar disorder with psychosis, while non‐affective psychosis included schizophrenia, schizoaffective disorder, schizophreniform, psychosis not otherwise specified (NOS), delusional disorder, or brief psychotic disorder. Participants were excluded from the HCP‐EP study for various reasons, including: (1) substance induced psychosis or psychosis due to a medical problem, (2) IQ less than 70, (3) active medical condition affecting the brain or cognitive functioning (e.g., seizure disorder, epilepsy, stroke), (4) severe substance use disorder within 90 days of screening, (5) high‐risk status for suicide (i.e., active ideation or suicide attempt within 30 days of screening), (6) electroconvulsive therapy (ECT) within 12 months of screening, (7) human immunodeficiency positive (HIV+) status, (8) contraindication to undergo MRI scan (e.g., implanted pacemaker, medication pump) and (9) overtly aggressive behaviour.

### 2.2 Measurement

Clinical assessments and questionnaires (including demographic questions) were administered by trained study personnel. The Structured Clinical Interview (SCID‐5‐RVl; First et al. 2014) was used to screen participants to establish eligibility to participate, and the following variables were assessed at baseline and are of focus in the current study:

#### 2.2.1 Suicide Ideation

Suicide ideation was measured using a single item of the Montgomery‐Asberg Depression Rating Scale (MADRS; Montgomery and Asberg 1979). The suicide item represents the feeling that life is not worth living, that a natural death would be welcome, suicide thoughts, and preparations for suicide. Response categories of this clinician administered scale range from 0 to 6 with no suicide thoughts being represented by a 0 and suicide plan with active preparations being represented by a 6. Item scores range from 0 to 6 with higher scores indicating more suicide ideation and movement towards plans and preparations.

#### 2.2.2 Depression

Depression was assessed with the MADRS, which uses 9 of the 10 items to measure sadness, tension, sleep, concentration, pessimistic thoughts, low energy and apathy. The suicide item was not included in the calculation of depression given its utilisation as an endogenous mediation variable. Response categories of this rating scale range from 0 to 6 and vary in alignment with each item (e.g., no sadness being represented by a 0 and extremely despondent being represented by a 6 for question 1 on apparent sadness). Total scores range from 0 to 54 with higher scores indicating more severe depression. Reliability analyses indicated minimal change from the one‐item removal with a Cronbach's alpha of 0.88 for the 10‐item scale and 0.87 for the 9‐item scale.

#### 2.2.3 Psychosis Symptoms

Psychosis symptoms were measured with the Positive and Negative Syndrome Scale (PANSS; Kay et al. 1987). The PANSS contains 30 items that assess for symptoms of psychosis including positive symptoms (e.g., hallucinations), negative symptoms (e.g., affective flattening) and general psychopathology (symptoms that are typically nonspecific to psychosis, yet still associated with psychosis in this population such as depression, anxiety, somatic concerns, impaired clinical insight, poor impulse control and more). Response categories range from absent (1) to extreme (7) and items are summed to obtain a total score. Positive symptom subscale scores range from 7 to 49, negative symptoms from 7 to 49 and general symptoms from 16 to 112; all with higher scores indicating greater presence and severity of symptoms.

#### 2.2.4 Distress

Distress was measured with the Patient‐Reported Outcomes Measurement Information System (PROMIS) emotional distress scale (Pilkonis et al. 2011). Item examples include ‘in the past 7 days I felt worried’ and ‘in the past 7 days I felt fearful’. Seven items of the scale focus on anxiety in relation to distress, with response categories ranging from 1 (never) to 5 (always) and higher total scores indicating the experience of greater distress.

### 2.3 Quantitative Modelling and Analysis

Data were analysed using SPSS 28 and Mplus 8 (Muthén and Muthén 1998–2017). Univariate distributions, bivariate correlations and missing data were examined among all variables. Latent Profile Analysis (LPA) was carried out to identify the latent profiles based upon 3 different subscale scores which capture positive, negative and general symptoms within psychosis. The number of profile groupings was determined by the model with the lowest Bayesian Information Criterion (BIC) and Akaike Information Criterion (AIC). Log likelihood ratio tests were also used to check for significantly better fitting models.

After LPA groupings were established, structural equation modelling (SEM) was performed to test a mediation model with multiple group analysis (moderation) using a robust (Huber‐White) maximum likelihood algorithm to deal with nonnormality and variance heterogeneity. Full Information Maximum Likelihood (FIML) methods were used, and model fit was evaluated using both global (χ^2^, CFI, TLI, standardised RMR, RMSEA) and focused (standardised residuals and modification indices) fit indices (Muthén and Muthén 1998–2017). Acceptable fit was determined by a minimum cutoff of 0.95 for CFI and TLI, a maximum cutoff of 0.06 for RMSEA and a maximum cutoff of 0.08 for SRMR (Kelloway 2014). Endogenous variables included suicide ideation (outcome variable), depression (mediation variable) and distress (mediation variable). Exogenous variables included positive symptoms, negative symptoms and general symptoms of psychosis. Sex, age and affective status (affective psychosis vs. non‐affective psychosis) were included as covariates in the model given their well‐documented relationships with suicide ideation in the literature (Beghi et al. 2021; Bertelsen et al. 2007; Björkenstam et al. 2014; Dutta et al. 2011; Fazel and Runeson 2020a, 2020b; Robinson et al. 2010).

## 3 Results

Demographic and clinical characteristics are presented in Table 1. Participants were 22.98 years of age (Standard Deviation (SD) = 3.57), on average, with a range of 16 to 34. The majority of participants were male (*n* = 102, 61.4%), White (*n* = 90, 54.2%) and non‐Hispanic/Latinx (*n* = 151, 91%). On average, participants attended 14.15 years of school (SD = 2.04) with a range of 10 (9th grade being the first year of high school) to 21 (4 years of graduate school) years. Most participants had a primary diagnosis of SSDs (*n* = 113, 68%), with the majority meeting criteria for schizophrenia (*n* = 70, 52.4%). Aside from SSDs, participants most often had bipolar disorder with psychotic features (*n* = 41, 24.7%) and depressive disorder with psychotic features (*n* = 10, 6%). Less than half of participants (*n* = 54, 32%) were categorised as having affective psychosis. Participants were on average exposed to antipsychotic medication for 12.43 months (SD = 14.50), were on a current antipsychotic medication dose of an average 102.71 mg (SD = 145), and were on a current clozapine equivalent dose on average of 156.10 (SD = 221.32), Lastly, the average suicide ideation continuous item score was 0.38 (SD = 0.91) with a range of 0 (no ideation) to 4 (ideation without specific plan or intent). Of the full sample, 18% (*n* = 30) endorsed having current suicide ideation.

**TABLE 1:** Characteristics of the full sample and by latent profile groups.

Characteristic	Full sample (*n* = 166)		Latent profile groupings					
			Group 1 (*n* = 114)		Group 2 (*n* = 29)		Group 3 (*n* = 23)	
	*n*	% or M ± SD	*n*	% or M ± SD	*n*	% or M ± SD	*n*	% or M ± SD
Age (M ± SD)	166	22.98 ± 3.57	114	22.94 ± 3.66	29	23.40 ± 3.68	23	22.67 ± 3.00
Years of education (M ± SD)	166	14.15 ± 2.04	57	14.26 ± 2.14	7	13.00 ± 1.63	8	14.37 ± 1.41
Sex								
Male	102	61.4	70	61.4	14	48.3	18	78.3
Female	64	38.6	44	38.6	15	51.7	5	21.7
Race								
Black or African American	56	33.7	34	29.8	11	37.9	11	47.8
White	90	54.2	66	57.9	16	55.2	8	34.8
American Indian or Alaska Native	2	1.2	1	0.9	0	0	1	4.3
Asian	10	6	6	5.3	1	3.4	3	13
More than one race	7	3.2	6	5.3	1	3.4	0	0
Ethnicity								
Hispanic/Latinx	15	9	8	7	4	13.8	3	13
Non‐Hispanic/Latinx	151	91	106	93	25	86.2	20	87
Affective psychosis status								
Affective psychosis	51	30.7	41	36	6	20.7	4	17.4
Non‐affective psychosis	115	69.3	73	64	23	79.3	19	82.6
Primary diagnosis								
Schizophrenia	79	52.4	48	42.1	15	48.3	16	69.6
Schizophreniform	10	6.1	5	4.4	3	10	2	8.7
Schizoaffective	17	10.2	11	9.6	5	16.7	1	4.3
Delusional disorder	2	1.2	2	1.8	0	0	0	0
Brief psychotic disorder	2	1.2	2	1.8	0	0	0	0
Psychosis NOS	3	3	5	4.4	0	0	0	0
Major depressive disorder with psychosis symptoms	10	6	7	6.1	2	6.9	1	4.3
Bipolar disorder with psychosis symptoms	41	24.7	34	29.8	4	13.8	3	13.0
Antipsychotic medication exposure								
None	9	8.9	4	6.2	5	23.8	0	0
Less than or equal to 1 month	9	8.9	6	9.2	2	9.5	1	6.7
More than 1 month and under 6 months	40	39.6	23	35.4	11	52.4	6	40
More than 12 months and less than or equal to 24 months	20	19.8	14	20	1	4.8	6	40
More than 24 months and less than or equal to 60 months	23	22.8	19	29.2	2	9.5	2	13.1
Months exposed to antipsychotic drug (M ± SD)	165	12.43 ± 14.50	114	12.94 ± 14.40	28	10.75 ± 14.41	23	11.96 ± 15.57
Current antipsychotic medication Mg dose (M ± SD)	115	102.71 ± 145.00	77	94.45 ± 155.09	19	116.37 ± 140.34	19	122.53 ± 104.96
Current clozapine equivalent dose	164	156.10 ± 221.32	113	117.70 ± 184.43	28	201.79 ± 224.63	23	289.13 ± 314.77
Clinical scales								
Depression (M ± SD)	114	7.95 ± 8.29	114	6.87 ± 7.03	29	15.00 ± 10.48	23	7.60 ± 11.24
Positive symptoms of psychosis (M ± SD)	114	11.50 ± 4.45	114	9.98 ± 2.93	29	18.62 ± 4.02	23	10.56 ± 3.08
Negative symptoms of psychosis (M ± SD)	114	13.28 ± 5.39	114	10.97 ± 3.18	29	13.79 ± 3.80	23	23.21 ± 3.44
General symptoms of psychosis (M ± SD)	114	24.63 ± 5.89	114	22.50 ± 4.25	29	32.24 ± 6.10	23	26.69 ± 4.49
Distress (M ± SD)	114	29.56 ± 6.74	114	28.97 ± 6.26	29	32.52 ± 8.59	23	28.70 ± 5.78
Degree of Suicide ideation (continuous; M ± SD)	114	0.38 ± 0.91	114	0.31 ± 0.821	29	0.63 ± 1.09	23	0.30 ± 1.02
Prevalence of suicide ideation (binary)	30	18.1	17	14.9	11	36.7	2	8.7

### 3.1 LPA

**FIGURE 1:**
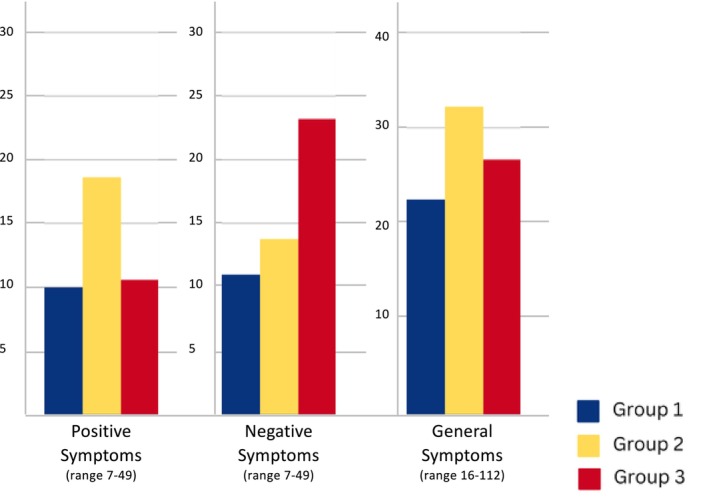
Mean scores of psychosis symptom subscales by LPA group.

A series of four models were estimated for the LPA specifying two through five profile groups with three psychosis variables (positive, negative and general symptoms). The three‐profile model had the lowest BIC and AIC values, with significantly better fit than the two‐, four‐ and five‐profile models. In addition to poor fit, one of the groups of the four‐ and five‐profile models included a sample size of 1. The final model with the best fit resulted in three groups: group 1 (G1) had 114 participants (68.6%), group 2 (G2) had 29 (17.5%) and group 3 (G3) had 23 (13.9%). LPA groups are displayed in Figure 1 where it can be observed that G1 had relatively lower and more balanced levels of symptoms overall (a similar amount of all three symptom areas) than the other groups, G2 had the highest positive and general symptoms than the other groups and G3 had the highest negative symptoms than the other groups.

Characteristics of LPA groups are also presented in Table 1. Participants were more often male in G1 (61.4%) and G3 (78.3%), and more often Black/African American in G2 (37.9%) and G3 (47.8%). As for clinical characteristics, G1 had the largest (36%) and G3 had the smallest (17.4%) prevalence of affective psychosis. In addition, a diagnosis of schizophrenia was most prevalent in G3 (69.6%), schizoaffective disorder in G2 (16.7%), major depressive disorder with psychosis symptoms in G1 (29.8%) and bipolar disorder with psychosis symptoms (29.8%). Participants in G1 were exposed to the longest duration of antipsychotic drugs (12.94 months), G3 was on the largest dosage of a current antipsychotic medication (112.53 mg) and G3 was also on the largest current clozapine equivalent dose (289.13 mg). As for suicide ideation, 14.9% endorsed having suicide ideation in G1, 36.7% in G2, and 8.7% in G3.

### 3.2 Multiple Group Analysis Model Fit and R^2^

**FIGURE 2:**
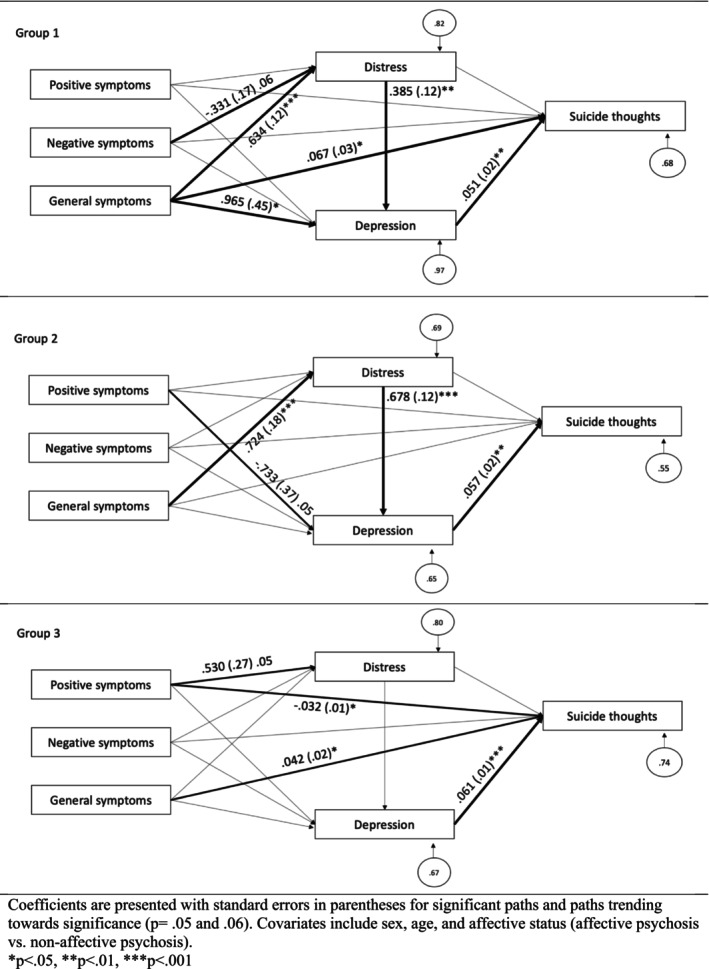
Model findings by LCA profile grouping.

Figure 2 presents parameter estimates for each LPA grouping of the mediation model with standard errors in parentheses. Global fit indices all pointed to good model fit (χ^2^ = 32.50, df = 27, *p* value = 0.214; CFI = 0.978, TLI = 0.948, RMSEA = 0.060, *p* value for close fit = 0.386, standardised RMR = 0.058) and focused fit indices (standardised residuals < ∣2∣ and modification indices < ∣4∣) revealed no points of stress on the model. For G1, positive symptoms, negative symptoms, general symptoms, distress, depression and covariates accounted for 32% of the variance in suicide ideation. For G2, positive symptoms, negative symptoms, general symptoms, distress, depression and covariates accounted for 45% of the variance in suicide ideation. For G3, positive symptoms, negative symptoms, general symptoms, distress, depression and covariates accounted for 26% of the variance in suicide ideation.

### 3.3 Direct Relationships by LPA Groups

For G1, general symptoms significantly related to distress, depression and suicide ideation. For every one‐unit increase in general symptoms, there was an independent associated average increase in distress (b = 0.634, Standard Error (SE) = 0.12, *p* < 0.001), depression (b = 0.965, SE = 0.45, *p* < 0.05) and suicide ideation (b = 0.067, SE = 0.03, *p* < 0.05). In addition, greater distress significantly related to increased depression (b = 0.385, SE = 0.12, *p* < 0.01) and greater depression to increased suicide ideation (b = 0.051, SE = 0.02, *p* < 0.01). The relationship between negative symptoms and distress was non‐significant, however trending (*p* = 0.06), in which greater negative symptoms associated with decreased distress (b = −0.331, SE = 0.17, *p* = 0.06).

For G2, general symptoms significantly related to distress, distress to depression and depression to suicide ideation. For every one‐unit increase in general symptoms, there was an associated average increase in distress (b = 0.724, SE = 0.18, *p* < 0.001). Greater distress significantly related to increased depression (b = 0.678, SE = 0.12, *p* < 0.001), and greater depression to increased suicide ideation (b = 0.057, SE = 0.02, *p* < 0.01). The relationship between positive symptoms and depression was non‐significant, however trending (*p* = 0.05), in which greater positive symptoms associated with decreased depression (b = −0.733, SE = 0.37, *p* = 0.05).

For G3, general symptoms significantly related to suicide ideation. For every one‐unit increase in general symptoms, there was an associated average increase in suicide ideation (b = 0.042, SE = 0.02, *p* < 0.05). In addition, greater positive symptoms related to decreased suicide ideation (b = −0.032, SE = 0.01, *p* < 0.05) and greater depression related to increased suicide ideation (b = 0.061, SE = 0.01, *p* < 0.001). The relationship between positive symptoms and distress was non‐significant, however trending (*p* = 0.05), in which greater positive symptoms associated with increased distress (b = 0.530, SE = 0.27, *p* = 0.05).

### 3.4 Mediation by LPA Groups

For G1, both distress and depression functioned as mediators in the model. Specifically, general symptoms related to suicide ideation both directly and indirectly through distress and depression with partial mediation being evident per the joint significance test (MacKinnon et al. 2002). Taken together as an indirect effect through distress and depression (general symptoms → distress → depression → suicide ideation), for every one‐unit increase in general symptoms, there was an average associated 0.012 unit increase in suicide ideation (*p* < 0.05). As an indirect effect through depression (general symptoms → depression → suicide ideation), for every one‐unit increase in general symptoms, there was an average associated 0.049 unit increase in suicide ideation (*p* < 0.05). As a total effect, for every one‐unit increase in general symptoms, there was an average 0.121 unit increase in suicide ideation (*p* < 0.01).

For G2, both distress and depression functioned as mediators in the model. Specifically, general symptoms related to suicide ideation indirectly through distress and depression with complete mediation being evident per the joint significance test (MacKinnon et al. 2002). Taken together as an indirect effect through distress and depression (general symptoms → distress → depression → suicide ideation), for every one‐unit increase in general symptoms, there was an average associated 0.028 unit increase in suicide ideation (*p* < 0.05). As a total effect, for every one‐unit increase in general symptoms, there was an average 0.063 unit increase in suicide ideation (*p* < 0.05).

For G3, distress and depression did not function as mediators in the relationships between psychosis symptoms and suicide ideation.

## 4 Discussion

Findings of the current study indicated that the relationships between positive, negative and general symptoms of psychosis, distress, depression and suicide ideation differed between latent groupings of psychosis symptoms. For G1 (lower and more balanced psychosis symptoms overall), general symptoms of psychosis directly related to distress, depression and suicide ideation. Distress also directly related to depression, and depression directly related to suicide ideation in this group. Of note, G1 had the largest prevalence of affective psychosis, major depressive disorder and bipolar disorder diagnoses, and were exposed to the longest duration of antipsychotic drugs other groups. For G2 (highest positive and general symptoms), general symptoms directly related to distress, distress directly related to depression and depression directly related to suicide ideation. For both G1 and G2, distress and depression functioned as mediators in the relationship between general symptoms and suicide ideation for those groups. Participants in G2 had the largest prevalence of schizoaffective disorder diagnosis and had the highest prevalence of suicide ideation than other groups. For G3 (highest negative symptoms), both positive and general symptoms significantly related to suicide ideation, and depression to suicide ideation. Interestingly, participants in G3 had the highest prevalence of schizophrenia diagnosis, were on the largest dosage of a current antipsychotic medication, and had the largest current clozapine equivalent dose than other groups.

In both G1 and G2, general symptoms played a large role in the relationships between psychosis symptoms and suicide ideation. Pertaining to the magnitude of relationships found, the coefficient for the total effect of general symptoms on suicide ideation through distress and depression was slightly greater for G1 (b = 0.121, *p* < 0.01) than for G2 (b = 063, *p* < 0.05). In comparing the two groups, those in G1 had a larger and more statistically significant total effect with overall fewer symptoms than those in G2, who had the highest positive and general symptoms of all groups. It is possible that those with fewer symptoms experienced less functional impairment and/or had stronger clinical insight, even if in the same phase of illness than those experiencing greater positive and general symptoms. Given prior research supports the role of distress on awareness of functional impairment and insight into illness and prognosis (Olvet et al. 2015; Phelan and Sigala 2022), more research is needed to elucidate the differential impact of distress in this population.

Alternatively, and given G1 were exposed to the longest duration of antipsychotic medication, it is possible that those with fewer symptoms (G1) were in a phase of illness in which they had been experiencing treatment and potential improvements in symptoms than those who had greater positive and general symptoms. Particularly so, as those in G2 had the shortest duration of antipsychotic medication exposure. Studies of symptom trajectories suggest a heterogeneous course for positive symptoms after psychosis onset with data of symptom reduction and stabilisation ranging from 1 to 10 years after onset (Austin et al. 2015; Birchwood, Todd, and Jackson 1998; Crumlish et al. 2009; Levine et al. 2011; Rabinowitz et al. 2007). Given such literature, it is likely that participants in the current study were in various stages of symptom experience within the first 5 years of onset and it is possible that some, and perhaps more people in G1, were in a phase of symptom reduction.

Greater negative symptoms of psychosis related to lower levels of distress in G1 as a non‐significant trend (*p* = 0.06). This negative symptom trend of distress aligns with prior research indicating that negative symptoms may relate less to distress (Holzworth et al. 2024; Griffiths et al. 2019; Vracotas et al. 2007); however, negative symptoms did not relate to distress in the group that had the most negative symptoms (G3). Increased negative symptoms may hinder one's ability to feel and/or identify distress, which could explain why some prior research has shown that negative symptoms do not relate with distress (Selten, Wiersma, and van den Bosch 2000). These findings highlight the mixed literature and gaps in knowledge about the impact of negative symptoms in the lives of individuals with psychosis, including the experience of psychological distress (Demyttenaere et al. 2022).

Positive symptoms of psychosis did not significantly relate to distress or depression in the model by LPA groups. There was, however, a trend (*p* = 0.05) of positive symptoms associating with decreased depression for the group with the highest positive and general symptoms (G2) and a trend (*p* = 0.05) of positive symptoms associating with increased distress for the group with highest negative symptoms (G3). These non‐significant, yet trending, positive symptom findings were not anticipated given the literature has established relationships between positive symptoms, distress and depression (Birchwood, Iqbal, and Upthegrove 2005; Saha et al. 2011). One potential rationale and mechanism is that social schemata may mediate the relationship between positive symptom experience and distress (Mawson, Cohen, and Berry 2010), and the current study did not measure or test social schemata. For example, the social appraisal of a positive symptom (such as a command auditory hallucination) being more or less powerful or threatening could result in different experiences in distress (Birchwood et al. 2000; Birchwood et al. 2004). Another potential rationale and mechanism is that certain positive symptoms can be experienced in conjunction with elevated mood states. For example, a manic phase in schizoaffective disorder, or an episode of grandiose delusions. These states could correlate negatively with depression and warrant further examination.

Consistent with prior literature, greater distress related to greater depression and subsequently higher levels of depression related to more suicide ideation (Freeman et al. 2015; Upthegrove, Marwaha, and Birchwood 2017). This aligns with Birchwood, Iqbal, and Upthegrove' (2005) proposed pathways to depression within psychosis in which an individual appraises the meanings and significance of their psychosis symptom experiences leading to the experience of depressive symptoms (Upthegrove, Marwaha, and Birchwood 2017). It is likely that distress may result from stress reactivity, challenges in emotion regulation and the self‐appraisal of factors like symptom experiences, diagnosis/diagnoses and cognitive and social functioning. This reinforces the findings of Martin and Colleagues (2015) that psychosis alone did not predict suicide attempts, but rather psychosis in combination with psychological distress became a strong predictor.

Overall, findings point towards two overarching implications: (1) suicide prevention efforts are critical in early psychosis populations and (2) there is need for greater focus on targeting depression and distress within intervention efforts of early psychosis populations. Although participants in the current study had relatively lower risk for suicide, given high suicide risk was exclusionary (i.e., suicide ideation or attempt within 30 days), nearly one in five experienced suicide thoughts shortly after screening (18%). This prevalence reinforces the knowledge that suicide thoughts and behaviours often fluctuate and is well‐aligned with literature suggesting that even in the absence of baseline ideation, the potential of subsequent suicide thoughts exists and warrant attention in this vulnerable population (Martínez‐Alés et al. 2023). As a result, it is important for providers to frequently assess for suicide risk and employ individualised approaches to suicide prevention, especially given depression and distress had different salience for specific psychosis symptom subgroups in the current study. Essentially, improving the tailoring of treatment for profiles of participants with more or fewer positive, negative and general symptoms will be important given variables related differently to distress, depression and suicide ideation. For example, an individual with more positive and general symptoms may benefit from greater focus on experiencing distress and depression, as compared to an individual who has more negative symptoms and less of an experience of distress and depression. Or perhaps, as discussed by Phelan and Sigala (2022), attention should be placed on skill building for the communication of distress to others given the greater challenges in organising and naming emotions in general among people with negative symptoms.

Relatedly, it is important to acknowledge challenges in clinical practice in detecting potentially small yet meaningful symptom experiences across patients in relation to suicide risk. This is important to note given some PANSS scores in the current study represented a level of mild or lower symptom experience, yet still significantly related to outcomes such as distress, depression and suicide ideation. This reinforces the well‐established literature of suicide risk being high in an early phase of psychosis illness with this being a critical period for treatment given the adjustment to onset, navigation of symptoms, stigma and more in addition to psychosis symptomatology (Ventriglio et al. 2016). It is essential that providers consider all individuals in an early phase of illness to be at heightened risk on their own and in addition to psychosis symptom acuity.

Pertaining to the second implication, it is essential to target depression and distress in treatment of individuals in an early phase of psychosis illness, especially as general symptoms of psychosis worsen. Psychotherapy treatments, such as Dialectical Behaviour Therapy (DBT) and Cognitive‐Behavioural Therapy (CBT), may be useful to address distress and depression among individuals in an early stage of psychosis illness. Although there is strong evidence in DBT's utility in suicide prevention (DeCou, Comtois, and Landes 2019; Lawlor et al. 2022; Mann, Michel, and Auerbach 2021; McCauley et al. 2018), few studies have investigated the intervention among psychosis samples, warranting future research. CBT‐specific suicide prevention‐focused approach with tailoring for individuals with psychosis, entitled Cognitive‐Behavioural Suicide Prevention for psychosis (CBSPp; Bornheimer et al. 2023; Bornheimer et al. 2024; Haddock et al. 2016; Pratt et al. 2010; Tarrier et al. 2013; Tarrier et al. 2013), may be useful to target distress and depression in suicide prevention.

Limitations of the current study are important to note. First, data were secondary in nature resulting in constraints, as the HCP‐EP was not designed to address the specific aims of the current study. Pertaining to sampling constraints, it is important to note that findings may not be generalizable to all individuals at risk for suicide given the HCP‐EP study excluded participants with high suicide risk status (i.e., active ideation or suicide attempt within 30 days of screening). High risk status being exclusionary is unfortunately common in psychosis‐specific research (Chalker et al. 2024), however, and importantly, 18% of our sample did endorse ideation soon after screening where such thoughts would have been exclusionary for participation. This reaffirms the dynamic nature of suicide risk and the importance of frequent risk assessment in this population. Second, and related to measurement constraints, future research should incorporate a distinct suicide‐related assessment measure as opposed to the use of items from within a scale. Also related to measurement, there were variables of importance that were not available for examination in the dataset yet could contextualise clinical profiles of LPA groupings and may relate differently to suicide risk across LPA groups. Variables include duration of untreated psychosis, psychosocial treatment engagement, hospitalisation history, schizoaffective disorder type (manic, depressive, mixed) and more. Third, the sample size was relatively small (*n* = 166), and especially so with LPA groupings of 114, 29 and 23 participants. Therefore, results are preliminary and future investigations are needed with larger samples to further examine psychosis LPA groupings and suicide outcomes. Lastly, the current study examined baseline data and therefore is cross‐sectional in nature; as such, causality and temporality cannot be established. Future longitudinal research should examine fluctuations in variables and their relationships with one another across time to establish the order of symptom experience (i.e., depressive symptoms prior to psychosis and vice versa).

In sum, the current study established latent profile groupings of psychosis symptom experiences and examined differences in a mediation model testing the relationships between psychosis symptoms, distress, depression and suicide ideation by group in a sample of individuals experiencing an early phase of psychosis. A better understanding of the roles that distress and depression play in the relationships between psychosis symptoms and suicide ideation can help inform modifiable targets of early intervention and subsequently decrease risk for suicide. Future research is critical to examine fluctuations in symptoms and suicide risk factors over time for individuals in an early phase of psychosis illness given the disproportionately high rates of suicide death in comparison to the general and other serious mental illness populations.

## Conflicts of Interest

The authors declare no conflicts of interest.

## Data Availability

The data that support the findings of this study are openly available in National Institute of Mental Health at https://nda.nih.gov/study.html?id=1279, reference number DOI: https://doi.org/10.15154/1522899.
